# Digits Across Languages: The Role of Lexical and Numerical Characteristics in Digit Span Performance Across Fourteen Languages

**DOI:** 10.1002/alz70857_106101

**Published:** 2025-12-25

**Authors:** Boon Lead Tee, Jee Eun Sung, Stefano F Cappa, Giovanni Carlesimo, Didem Öz, Yağmur Özbek, Görsev Yener, Suvarna Alladi, Faheem Arshad, Avanthi Paplikar, Maxime Montembeault, Fernando Henriquez, Andrea Slachevsky, Adolfo M Garcia, Guerry M. Peavy, Clara Li, Serggio Lanata, Nevine El Nahas, Tamer Roushdy, Isabel Elaine Allen, Gloria A Aguirre, Guan Xue Chen, Xin Wen, Maria Luisa Gorno Tempini

**Affiliations:** ^1^ Global Brain Health Institute, University of California, San Francisco, San Francisco, CA, USA; ^2^ Memory and Aging Center, UCSF Weill Institute for Neurosciences, University of California, San Francisco, San Francisco, CA, USA; ^3^ Ewha Womans University, Seodaemun‐gu, Seoul, Korea, Republic of (South); ^4^ IUSS, Pavia, Italy; ^5^ IRCCS Mondino Foundation, Pavia, Italy; ^6^ IRCCS S. Lucia Foundation, Rome, Rome, Italy; ^7^ Department of Systems Medicine, Tor Vergata University, Rome, Italy; ^8^ GBHI ‐ UCSF, San Francisco, CA, USA; ^9^ Dokuz Eylul University Hospital Neurology Department, Izmir, Turkey; ^10^ Department of Neurosciences, Health Science Institute, Dokuz Eylül University, Izmir, Turkey; ^11^ Dokuz Eylul University, Izmir, Turkey; ^12^ Dokuz Eylül University, Balçova, Izmir, Turkey; ^13^ Izmir University of Economics, Faculty of Medicine, Balçova, Izmir, Turkey; ^14^ National Institute of Mental Health and Neurosciences [NIMHANS], Bengaluru, Karnataka, India; ^15^ Global brain Health institute, California, CA, USA; ^16^ National Institute of Mental Health and Neurosciences, Bengaluru, India; ^17^ Dr. S. R. Chandrasekhar Institute of Speech and Hearing, Bengaluru, India; ^18^ University of Montreal, Montreal, QC, Canada; ^19^ Douglas Mental Health University Institute, Montréal, QC, Canada; ^20^ Neuropsychology and Clinical Neuroscience Laboratory (LANNEC), Physiopathology Department ‐ ICBM, Neuroscience and East Neuroscience Departments, Faculty of Medicine, Universidad de Chile, Santiago, Metropolitana, Chile; ^21^ Interdisciplinary Center for Neuroscience (NeuroUC) ‐ Laboratory for Cognitive and Evolutionary Neuroscience ‐ Medicine School ‐ Pontificia Universidad Católica de Chile, Santiago, Chile; ^22^ Geroscience Center for Brain Health and Metabolism (GERO), Santiago, Chile; ^23^ Memory and Neuropsychiatric Center (CMYN) Neurology Department, Hospital del Salvador and Faculty of Medicine, University of Chile, Santiago, Region Metropolitana, Chile; ^24^ Neuropsychology and Clinical Neuroscience Laboratory (LANNEC), Physiopathology Department ‐ ICBM, Neuroscience and East Neuroscience Departments, Faculty of Medicine, Universidad de Chile, Santiago, Chile; ^25^ Servicio de Neurología, Departamento de Medicina, Clínica Alemana‐Universidad del Desarrollo, Santiago, Chile; ^26^ Cognitive Neuroscience Center (CNC), Universidad de San Andrés, Buenos Aires, Buenos Aires, Argentina; ^27^ Departamento de Lingüística y Literatura, Facultad de Humanidades, Universidad de Santiago de Chile, Santiago, Chile; ^28^ Global Brain Health Institute (GBHI), University of California San Francisco (UCSF); & Trinity College Dublin, Dublin, Ireland; ^29^ Department of Neurosciences, University of California San Diego, San Diego, CA, USA; ^30^ Icahn School of Medicine at Mount Sinai, New York, NY, USA; ^31^ Global Brain Health Institute, San Francisco, CA, USA; ^32^ Neurology Department, Faculty of Medicine, Ain Shams University, Cairo, Cairo, Egypt; ^33^ Department of Epidemiology and Biostatistics, University of California, San Francisco, San Francisco, CA, USA; ^34^ Global Brain Health Institute, University of California, San Francisco, CA, USA, San Francisco, CA, USA, San Francisco, CA, USA; ^35^ Memory and Aging Center, UCSF Weill Institute forNeurosciences, University of California, San Francisco, San Francisco, CA, USA, San Francisco, CA, USA; ^36^ Memory and Aging Center, University of California San Francisco, San Francisco, CA, USA; ^37^ Memory and Aging Center, University of California San Francisco, SAN FRANCISCO, CA, USA; ^38^ Department of Neurology, Memory and Aging Center, University of California San Francisco, San Francisco, CA, USA

## Abstract

**Background:**

The digit span task, a measure of auditory verbal short‐term and working memory, is widely used globally. Emerging research has revealed variations in digit span performance across languages among young adults; however, studies focusing on older populations are scarce and typically involving limited languages. This study investigates digit span performance among older adults (40‐90 year‐old) across fourteen languages and explored the influence of lexical and numerical properties on cognitive assessment.

**Method:**

We examined digit span performance among cognitively normal participants (CN), individuals with mild cognitive impairment (MCI) and Alzheimer's disease (AD) across fourteen language cohorts totalling 3,681 participants: English (*n* = 446), Mandarin (*n* = 97), Cantonese (*n* = 65), Spanish (*n* = 218), Kannada (*n* = 69), Hindi (*n* = 72), Telugu (*n* = 69), Malayalam (*n* = 70), Bengali (*n* = 70), French (*n* = 299), Korean (*n* = 1098), Italian (*n* = 540), Arabic (*n* = 50), and Turkish (*n* = 518). First, we analyzed language differences in digit span performance among CN using ANOVA and general linear models. We then conducted Receiver operating characteristic (ROC) analyses to identify the optimal cutoff values for AD. Next, we computed the digit count, syllable count, and numerical magnitude (i.e. the average sum of the digits) of all digit stimuli in the English cohort and analyzed their effects via linear and ridge regression analyses.

**Result:**

The forward (FDS) and backward digit span (BDS) tests revealed significant differences among CN across the fourteen language cohorts even after adjusting for age and education (FDS:F=38.62, *p* <0.001; BDS:F=19.23, *p* <0.001). ROC analysis revealed varying optimal cutoff values across languages: English (FDS:6, BDS:4), Italian and Turkish (FDS:5, BDS:4), Mandarin (FDS:7, BDS:5), Cantonese (FDS:7, BDS:4), and French (FDS:6, BDS:3). Further analysis indicated that the interaction between digit and syllable counts significantly impacted FDS accuracy in English speakers (linear:p=0.00035; ridge:p< 0.000001), with no significant effect from digit count alone after adjusting for interaction. Conversely, BDS performance showed a significant negative influence from digit count (*p* = 0.00858), with numerical magnitude and syllable count nearing significance (*p* = 0.083 and *p* = 0.066, respectively).

**Conclusion:**

Variations in digit span performance across languages illustrate the role of linguistic and numerical factors in cognitive assessments, even with tests targeting non‐language domains using digit stimuli. These findings underscore the critical value of language diversity in cognitive research.

